# Paradoxical Effects of Performance Pressure on Employees’ In-Role Behaviors: An Approach/Avoidance Model

**DOI:** 10.3389/fpsyg.2021.744404

**Published:** 2021-10-22

**Authors:** Xiaofeng Xu, Yihui Wang, Miaomiao Li, Ho Kwong Kwan

**Affiliations:** ^1^Department of Business Administration, School of Economics and Management, Tongji University, Shanghai, China; ^2^College of Management and Economics, Tianjin University, Tianjin, China; ^3^School of Politics and Public Administration, Qinghai Minzu University, Xining, China; ^4^Organizational Behavior and Human Resource Management Department, China Europe International Business School (CEIBS), Shanghai, China

**Keywords:** performance pressure, self-objectification, workplace anxiety, in-role behaviors, work meaningfulness, approach/avoidance motivation

## Abstract

Performance pressure acts as a double-edged sword for employees. Based on an approach/avoidance framework, we theorize that performance pressure produces both positive and negative effects on employees’ in-role behaviors *via* approach motivation (i.e., self-objectification) and avoidance motivation (i.e., workplace anxiety), and work meaningfulness moderates employees’ reactions to performance pressure. We examine our hypotheses using data from a sample of 345 employees in various organizations. The results show that self-objectification provides an approach motive that mediates the positive indirect effect of performance pressure on employees’ in-role behaviors. However, workplace anxiety provides an avoidance motive that mediates the negative indirect effect of performance pressure on employees’ in-role behaviors. Work meaningfulness strengthens both the approach and avoidance tendencies that employees experience under performance pressure. Our findings have significant theoretical and managerial implications.

## Introduction

In the 21st century, organizations are facing increasingly complex, dynamic, and competitive business environments. For example, the coronavirus disease 2019 (COVID-19) pandemic has taken an unprecedented toll on global economic welfare (Horesh and Brown, 2020). Since the resumption of work, organizations have vigorously expanded production, seeking to recover their losses. They have also called on employees to expand their abilities and improve performance. Hence, employees have experienced increased performance pressure. Performance pressure, defined as “the urgency to achieve high-performance levels because performance is tied to substantial consequences” (Mitchell et al., 2019, p. 533; Mitchell et al., 2018), is one of the most critical factors in today’s workplace (Leinhos et al., 2018), and it has attracted increasing attention from numerous scholars (e.g., Gardner, 2012; Mitchell et al., 2019). Many scholars have recognized performance pressure as a double-edged sword, as it can trigger both helpful and harmful side effects for employees and organizations (e.g., Gardner, 2012; Gutnick et al., 2012; Mitchell et al., 2019). For instance, Mitchell et al. (2019) found that performance pressure can produce paradoxical effects of appraisal: a threatening appraisal and a challenging appraisal.

Although performance pressure clearly acts as a double-edged sword for employees, the process by which it affects different people in different ways remains poorly understood. Performance pressure can focus employees’ efforts on improved performance, but it is unclear whether increasing that pressure is a productive or unproductive strategy for generating beneficial work behaviors (Mitchell et al., 2019), such as improved adherence to traditional performance of in-role behaviors (Williams and Anderson, 1991). In-role behaviors refer to the ways that employees do their formally prescribed job responsibilities, such as complying with rules or regulations and completing assigned duties on time (Williams and Anderson, 1991; Turnley et al., 2003). Managers typically give these essential types of behavior the most weight when they rate an employee’s overall performance (Rotundo and Sackett, 2002). However, as a double-edged sword, performance pressure may facilitate or debilitate an employee’s in-role behaviors, depending on each employee’s reactions. To analyze this reaction process, we apply an approach/avoidance framework to develop a better understanding of the dual effect that performance pressure can have on employees’ in-role behaviors. An approach/avoidance framework is a basic or natural categorization scheme in which phenomena can be categorized according to whether they stimulate approach or avoidance motions (Ferris et al., 2016).

Based on an approach/avoidance model, we argue that performance pressure as a unique source of work stress may activate both an approach and avoidance motivation (i.e., self-objectification and workplace anxiety) toward the desired in-role behaviors. On the one hand, self-objectification, defined as the internalization of objectifying experiences (Frederickson and Roberts, 1997; Gruenfeld et al., 2008; Jones and Griffiths, 2015), is an approach to appraising one’s own goal-related behaviors and objectives in performance-related settings (Gruenfeld et al., 2008; Poon et al., 2020). Exposure to performance pressure, individuals practice self-objectification when they treat themselves as objects or tools for achieving instrumental goals, such as attaining performance goals (Jones and Griffiths, 2015; Poon et al., 2020). On the other hand, workplace anxiety called performance-based anxiety, defined as a feeling of nervousness, unease, or tension about job-related performance (McCarthy et al., 2016; Cheng and Mccarthy, 2018), is a prototypical motive for avoidance behavior (Lerner and Keltner, 2001; Ferris et al., 2016). Anxiety typically occurs under the threat-related stimuli that may be performance pressure (Mitchell et al., 2019). According to attentional control theory (Eysenck et al., 2007), anxiety tends to impair attentional control. More specifically, anxiety increases attention to performance pressure, subsequently reducing attentional focus on current task-relevant behaviors (Eysenck et al., 2007).

Because performance pressure can produce paradoxical effects on employees’ in-role behaviors, we seek to explain why some employees tend to intensify the approach and avoidance motivation in response to performance pressure. Faced with work performance, the degree to which employees see their work as meaningful differs according to each individual perspectives and experiences (Rosso et al., 2010). Work meaningfulness is the extent to which individuals feel that their work activities are generally significant, valuable, and purposeful (Hackman and Oldham, 1980; Rosso et al., 2010; Lysova et al., 2019). When employees face pressure to perform work that they view as meaningful, they may find it helpful to answer the question, “Why do I work so hard?” In that case, when they experience anxiety or self-doubt, they may attribute their problem to something else. However, employees who feel that their work is less meaningful may be less motivated to address performance pressure and less anxious about expected performance. Therefore, we argue that the perceived meaningfulness of work can moderate the relationship between performance pressure and in-role behaviors and that it does so through approach and avoidance responses.

**Figure 1:**
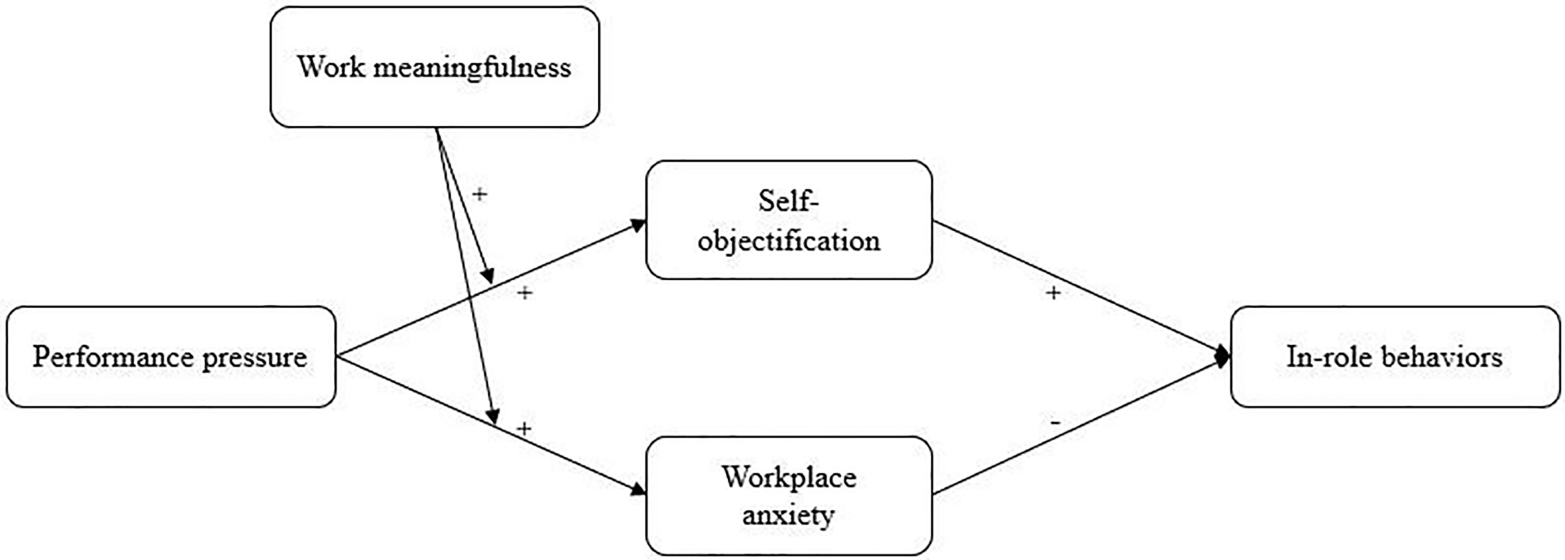
Hypothesized Model

Based on the above research impetus, the purpose of our study was to explore the double sides of performance pressure. This study applies an approach/avoidance framework to develop a theoretical model presented in Figure 1 and address the following research questions: (1) How does performance pressure influence on employees’ in-role behaviors *via* an approach (self-objectification) and avoidance motivation (workplace anxiety)? (2) How does work meaningfulness moderate the relationship between performance pressure, approach/avoidance motivation, and employees’ in-role behaviors.

The rest of the paper is organized as follows. “THEORY AND HYPOTHESES” elaborates the theory and hypotheses. “RESEARCH METHOD” addresses the methodology. “RESULTS” presents the results of the data analyses. “DISCUSSION and CONCLUSION” conclude the research, discuss findings, and provide some implications and suggestions for future research.

## Theory and Hypotheses

### Performance Pressure and Employees’ In-Role Behaviors

Performance pressure can act as a double-edged sword (Gardner, 2012; Gutnick et al., 2012; Mitchell et al., 2019), exerting both positive and negative side effects on employees’ in-role behaviors. Some observers have contended that employees can appraise performance pressure as both a challenge and as a threat stressor at the same time, and therefore, such pressure can engender both positive (e.g., engagement, task proficiency, and citizenship) and negative (e.g., self-regulation depletion and incivility) consequences (Gutnick et al., 2012; Mitchell et al., 2019). Given that studies have highlighted the positive and negative side effects of performance pressure, it is reasonable to assume that such pressure may have varied effects on employees’ in-role behaviors. Performance pressure introduces a sense of urgency to improve performance and achieve desirable outcomes (Lazarus, 2000). When employees experience performance pressure, they realize that meeting or exceeding the performance expectations can earn them raises, promotions, and other benefits (Mitchell et al., 2019). Consequently, many employees perform well in response to pressure. However, performance pressure is also a stressor for employees (Mitchell et al., 2019), and such stress can have negative effects on their in-role behaviors. Therefore, we consider how performance pressure can produce both positive and negative effects on employees’ in-role behaviors.

The processes by which performance pressure affects employees’ in-role behaviors remain unclear. To clarify these processes, we apply the approach/avoidance framework, which has been widely applied in explaining and predicting motivation and behavior (Elliot, 2006; Ferris et al., 2016). Elliot (2006) explained the difference between approach and avoidance motives as follows: “Approach motivation may be defined as the energization of behavior by, or the direction of behavior toward, positive stimuli (objects, events, possibilities), whereas avoidance motivation may be defined as the energization of behavior by, or the direction of behavior away from, negative stimuli (objects, events, possibilities)” (p. 112). The key point is that people are motivated to move toward positive stimuli and away from negative stimuli. In this sense, approach and avoidance motives produce positive and negative consequences. In using this approach/avoidance framework, we argue that approach and avoidance motives can play crucial roles in the relationship between performance pressure and employees’ in-role behaviors.

### An Approach/Avoidance Mediating Effect

In general, employees may have one of two distinct responses to performance pressure: to either approach the challenge or to avoid and move away from it. For those who tend to approach the challenge, we presume that performance pressure activates their approach motivation (i.e., their self-objectification in moving toward a challenge). Self-objectification is an approach to appraising one’s own goal-related behaviors and objectives (Gruenfeld et al., 2008). This approach involves an internalization of objectification, by which people treat themselves as instrumental objects or tools to achieve a performance goal (Gruenfeld et al., 2008; Jones and Griffiths, 2015; Poon et al., 2020). Performance pressure is focused on enhancing results, and employees can view this challenge as an opportunity to grow and achieve personal goals in career development (Mitchell et al., 2019). Individuals have an innate need to master their destiny and actualize their potential (Deci and Ryan, 2000). To meet high-performance expectations, employees tend to objectify themselves, because such objectification is a useful, instrumental tool for achieving performance goals (Gruenfeld et al., 2008; Poon et al., 2020). Therefore, we hypothesize the following:

*Hypothesis 1*: Performance pressure is positively related to self-objectification.

Performance pressure may also evoke tendencies to avoid stress and difficulty (i.e., workplace anxiety). Workplace anxiety, which is also called performance-based anxiety, is defined as a feeling of nervousness, tension, and worry about job-related performance (McCarthy et al., 2016; Cheng and Mccarthy, 2018). An employee in this condition views workplace anxiety as a prototypical motive for avoidance behavior (Lerner and Keltner, 2001; Ferris et al., 2016). Performance pressure arises from demands for improved results and the need for employees to constantly upgrade how they work, as they worry about whether their efforts can meet the performance goals (Rousseau, 1997; Dalal et al., 2009). In this respect, performance pressure is a threatening stressor (Cheng and Mccarthy, 2018; Mitchell et al., 2019), and employees are naturally prone to anxiety over their capacity to meet performance-related demands. Hence, we propose the following hypothesis:

*Hypothesis 2*: Performance pressure is positively related to workplace anxiety.

According to the principles of approach and avoidance (Elliot, 2006), approach motives are likely to stimulate positive behaviors and avoidance motives are likely to induce negative behaviors. In considering these motives, we argue that self-objectification enables employees to better engage in their in-role activities. According to objectification theory, self-objectification is a process in which individuals treat themselves as objects or tools for achieving instrumental goals (Frederickson and Roberts, 1997; Gruenfeld et al., 2008; Jones and Griffiths, 2015). In other words, self-objectification can help people achieve their aims. In this objectification process, employees treat performance pressure as a positive stimulus that can motivate them to do their in-role job better and meet their firms’ goals. Therefore, we hypothesize the following:

*Hypothesis 3*: Employees’ self-objectification is positively related to their in-role behaviors.

However, workplace anxiety can also seriously hamper an employee’s performance of in-role behaviors. According to attentional control theory (Eysenck et al., 2007), workplace anxiety can impair an individual’s attentional control. Anxious individuals typically pay close attention to threat-related stimuli. They worry and immerse themselves in negative concerns and therefore give inadequate attention to their current tasks. A mass of empirical studies have indicated that workplace anxiety is negatively associated with job performance (Ford et al., 2011; McCarthy et al., 2016). Therefore, it is reasonable to presume that workplace anxiety can hinder an employee’s in-role behaviors, and we propose the following hypothesis:

*Hypothesis 4*: Workplace anxiety is negatively related to employees’ in-role behaviors.

Consistent with the arguments developed above, we expect self-objectification and workplace anxiety to mediate the indirect effects of performance pressure on employees’ in-role behaviors. Therefore, we propose the following two hypotheses:

*Hypothesis 5*: Self-objectification mediates the positive indirect effect of performance pressure on employees’ in-role behaviors.

*Hypothesis 6*: Workplace anxiety mediates the negative indirect effect of performance pressure on employees’ in-role behaviors.

### The Moderating Role of Work Meaningfulness

Work meaningfulness is the degree to which employees regard their work as significant and purposeful (Rosso et al., 2010; Zhang et al., 2020). The sense of meaningfulness has been demonstrated to positively influence several kinds of work-related sentiments, such as motivation, engagement, job satisfaction, and so on (for a review, see Rosso et al., 2010). In addition, various scholars have proposed that work meaningfulness plays a moderating role in personal performance (Harris et al., 2007; Zhang et al., 2020). For instance, Zhang et al. (2020) found that work meaningfulness can strengthen the positive relationship between core self-evaluation and knowledge sharing, which can further facilitate creativity. However, Harris et al. (2007) suggested that work meaningfulness can also intensify the negative effects that abusive supervisors have on job performance.

In this study, we propose that work meaningfulness moderates the effects of performance pressure on approach and avoidance motivations (i.e., self-objectification and workplace anxiety). Spreitzer (1995) claimed that the process of finding meaning in work involves seeing a fit between the requirements of a work role and the worker’s beliefs, values, and behaviors. Thus, individuals hold different views of work meaningfulness in relation to their own work experiences (Rosso et al., 2010). When individuals experience a high level of meaning in their work, they tend to invest substantial resources, find a high level of fit between themselves and their jobs, and even experience a sense of ultimate purpose in their work (Spreitzer, 1995; Harris et al., 2007). As a result, when individuals who experience a high level of work meaningfulness are exposed to performance pressure, they are more likely to put forth their best efforts to meet the performance goals. They are also likely to worry about their standing in their organization (Harris et al., 2007). Therefore, they are more likely to both view themselves as instrumental tools (i.e., to practice self-objectification) for meeting performance goals and to experience more anxiety. However, when individuals perceive little meaning in their work, they are unlikely to find a good sense of fit with their jobs. They also tend to invest fewer resources in their work and to care less about meeting performance-related goals. Therefore, we propose the following two hypotheses:

*Hypothesis 7*: Work meaningfulness moderates the positive relationship between performance pressure and self-objectification, such that the positive relationship is stronger when employees perceive higher levels of work meaningfulness.

*Hypothesis 8*: Work meaningfulness moderates the positive relationship between performance pressure and workplace anxiety, such that the positive relationship is stronger when employees perceive higher levels of work meaningfulness.

Furthermore, we expect work meaningfulness to strengthen the positive indirect relationship between performance pressure and employees’ in-role behaviors through the process of self-objectification. And work meaningfulness may also strengthen the negative indirect relationship between performance pressure and employees’ in-role behaviors *via* workplace anxiety due to the significant negative side effect of this relationship. Therefore, we propose the following two hypotheses of moderated mediation:

*Hypothesis 9*: Work meaningfulness can strengthen the positive indirect relationship between performance pressure and employees’ in-role behaviors through self-objectification.

*Hypothesis 10*: Work meaningfulness can strengthen the negative indirect relationship between performance pressure and employees’ in-role behaviors through workplace anxiety.

## Research Method

### Research Approach

Our study used a quantitative research approach. We conducted online survey *via* WeChat and adopted a snowball sampling strategy to collect data, and the reasons are as follows. First, WeChat is a multipurpose messaging application with over 1billion active users (Qin et al., 2020), and it is a low-cost method to collect data. Second, the response rate is higher than manual distribution of a questionnaire (Rasool et al., 2021). Third, the data collection was conducted during the COVID-19 epidemic in China, that most of employees were observing the lockdown period or were working from home. Therefore, online survey *via* WeChat is an appropriate strategy to collect data.

### Instrument Development

In this research, we first designed the questionnaire for data collection, the questionnaire comprised demographic characteristics, five variables (i.e., performance pressure, work meaningfulness, self-objectification, workplace anxiety, and in-role behaviors), including 23 items scored with a 5-point Likert scale (1=strongly disagree and 5=strongly agree). These scales were initially developed in English and widely used with higher internal consistency and retest reliability. We translated them into Chinese in strict conformity with Brislin (1986) back-translation principles. Two researchers and one expert carried out the translation procedures.

### Data Collection and Sample

Next, three of the researchers disseminated our online survey to employees, and we encouraged the employees passed the questionnaire on to others and give a certain reward. Ultimately, a total of 373 responses were collected from various companies in China. After screening out data that were not properly entered, we retained a final sample of 345 participants (a valid response rate of 92.5%). This sample covered 57 cities in 20 provinces of China.

Of the 345 participating employees, 139 were male and 206 were female. Their education levels were relatively high, with 172 (49.9%) holding a Bachelor’s degree and 75 (21.7%) holding a Master’s degree or above. The participants worked in a variety of organizations, such as government departments, public institutions, foreign enterprises/joint ventures, state-owned enterprises, and private enterprises.

### Measures

The participants rated all of the measures by using a 5-point Likert scale, and we averaged these variables for data analysis.

#### Performance Pressure

We used the four-item scale developed by Mitchell et al. (2018). A sample item is “The pressures for performance in my workplace are high.” This scale’s Cronbach alpha was 0.8.

#### Work Meaningfulness

We adopted the three-item scale developed by Spreitzer (1995). A sample item is “The work I do is very important to me.” This scale’s Cronbach alpha was 0.9.

#### Self-Objectification

We adapted the three-item scale from Poon et al. (2020). The items are “I feel objectified,” “I feel like I am being treated as an object,” and “People treat me as a tool.” This scale’s Cronbach alpha was 0.6.

#### Workplace Anxiety

We used the eight-item scale from McCarthy et al. (2016), which was modified from the performance anxiety scale developed by McCarthy and Goffin (2004). A sample item is “I am overwhelmed by thoughts of doing poorly at work.” This scale’s Cronbach alpha was 0.9.

#### In-Role Behaviors

We adapted a five-item scale from Huang and Hsieh (2015), which was adapted from Williams and Anderson (1991). A sample item is “I can adequately complete assigned duties.” This scale’s Cronbach alpha was 0.9.

#### Control Variables

We controlled for the participants’ demographics, including gender (1=male, 2=female), age (1=under 25years, 2=between 25 and 30years, 3=between 31 and 35years, 4=between 36 and 40years, 5=between 41 and 45years, 6=between 46 and 50years, and 7=above 50years), education (1=high school or below, 2=associate degree, 3=bachelor’s degree, and 4=master’s degree or above), organization type (1=government departments, 2=public institutions, 3=foreign enterprises/joint ventures, 4=state-owned enterprises, 5=private enterprises, and 6=others), organizational tenure (1=under 1year, 2=between 1 and 2years, 3=between 3 and 5years, 4=between 5 and 7years, and 5=above 7years), and position (1=frontline employee, 2=frontline managers, 3=middle managers, and 4=senior managers).

## Results

### Common Method Bias Test

According to Podsakoff et al. (2003), we examined the potential for common method bias by using a statistical procedure. Using an orthogonal method factor and calculating the average of the squared loadings on the common method factor, the results were 11.39%, lower than the 17.2% reported by Williams and McGonagle (2016). Thus, common method variance (CMV) was not a serious issue.

### Confirmatory Factor Analyses

We conducted confirmatory factor analyses (CFA) to examine the distinctiveness of the five variables (i.e., performance pressure, work meaningfulness, self-objectification, workplace anxiety, and in-role behaviors). The five proposed factors have better fit indices (*χ*^2^=536.07, *p*<0.001, df=199, CFI=0.92, TLI=0.91, RMSEA=0.07, and SRMR=0.07) than alternative models, such as the four-factor model with self-objectification and workplace anxiety combined (*χ*^2^=714.47, p<0.001, df=203, CFI=0.88, TLI=0.86, RMSEA=0.09, and SRMR=0.08). Therefore, the discriminant validity of the proposed five-factor model was confirmed.

### Descriptive Statistics and Correlations

All of the variables’ means, standard deviations, and correlations are presented in Table 1.

**Table 1:** Means, standard deviation, and correlations among variables.

Variable	M	S.D.	1 (Pearson Chi-square)	2	3	4	5	6	7	8	9	10	11
1. Gender (0=male, 1=female)	0.60	0.49											
2. Age	2.08	1.07	7.05										
3. Education	2.88	0.81	5.49	−0.06									
4. Organizational type	3.72	1.88	9.82	−0.19^***^	−0.11								
5. Organizational tenure	2.33	1.21	7.33	0.59^***^	−0.04	−0.19^***^							
6. Position	1.41	0.71	15.78^**^	0.29^***^	−0.03	0.06	0.39^***^						
7. Performance pressure	3.36	0.78	20.16	0.05	−0.03	0.06	−0.10	0.003	*(0.8)*				
8. Work meaningfulness	3.71	0.86	9.47	0.18^**^	0.08	−0.13^*^	0.09	0.05	0.15^**^	*(0.9)*			
9. Self-objectification	3.27	0.63	14.16	−0.003	0.11^*^	−0.07	0.10	−0.04	0.28^***^	0.18^**^	*(0.6)*		
10. Workplace anxiety	3.29	0.87	28.63	−0.03	−0.03	−0.03	−0.13^*^	−0.12^*^	0.66^***^	0.02	0.31^***^	*(0.9)*	
11. In-role behaviors	3.76	0.65	26.46	0.17^**^	0.14^**^	−0.03	0.09	0.09	0.01	0.41^***^	0.13^*^	−0.09	*(0.9)*

*n=* 345 Cronbach’s alpha is in parentheses.

*** *p<* 0.001;

** *p<* 0.01;

* *p<* 0.05.

### Hypothesis Testing

#### Direct and Indirect Effects

We use structural equation modeling (SEM) through MPLUS 7.4 with a bootstrapping technique (bootstrap=1,000) to test the direct and indirect effects. Table 2 shows the results of the direct and indirect effects as following. Performance pressure was positively related to self-objectification (*β*=0.19, *p*<0.05) and workplace anxiety (*β*=0.75, *p*<0.001), thus supporting hypotheses 1–2. Self-objectification was positively related to employees’ in-role behaviors (*β*=0.30, *p*<0.001), and workplace anxiety was negatively related to employees’ in-role behaviors (*β*=−0.20, *p*<0.05), thus supporting hypotheses 3–4. The indirect effect of performance pressure on employees’ in-role behaviors through self-objectification was significant and positive (*β*=0.06, *p*<0.05) and that the indirect effect of performance pressure on employees’ in-role behaviors through workplace anxiety was significant and negative (*β*=−0.15, *p*<0.05), thus supporting hypotheses 5–6.

**Table 2:** Direct and indirect effects.

	Estimate	S.E.	*p*-values
*Direct paths*			
P→SO	0.19	0.08	0.022
P→WA	0.75	0.08	0.000
SO→IRB	0.30	0.07	0.000
WA→IRB	−0.20	0.09	0.026
P→IRB	0.08	0.10	0.435
*Indirect paths*			
P→SO→IRB	0.06	0.03	0.049
P→WA→IRB	−0.15	0.07	0.032

P, performance pressure; SO, self-objectification; WA, workplace anxiety; IRB, in-role behaviors.

#### Moderating Effects

**Figure 2:**
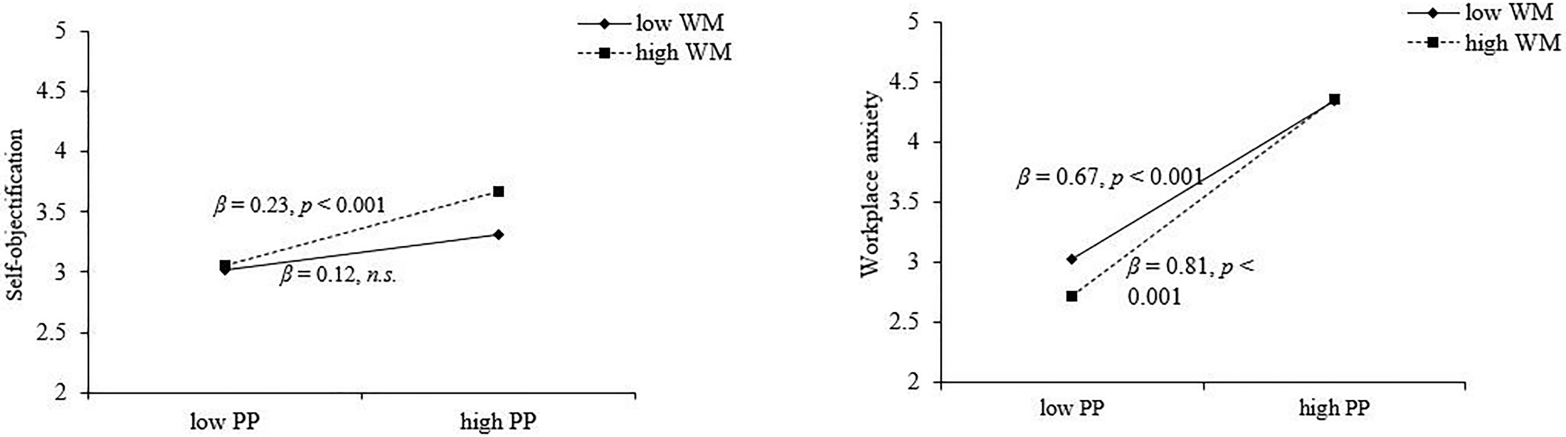
The interaction between performance pressure (PP) and work meaningfulness (WM) on self-objectification and workplace anxiety.

We conducted ordinary least square regression analyses by using SPSS 23.0 to test the moderating role of work meaningfulness. Table 3 and Figure 2 show that work meaningfulness significantly moderated the association between performance pressure and self-objectification (*β*=0.08, *p*<0.05). In other words, the positive relationship between performance pressure and self-objectification was reinforced when the level of work meaningfulness was higher (*β*=0.23, *p*<0.001) rather than lower (*β*=0.12, *n. s.*), hence supporting hypothesis 7. In addition, the moderating effect of work meaningfulness was marginally significant (*β*=0.08, *p*=0.05), indicating that the positive relationship between performance pressure and workplace anxiety was strengthened when the level of work meaningfulness was higher (*β*=0.81, *p*<0.001) rather than lower (*β*=0.67, *p*<0.001). Thus, hypothesis 8 was marginally supported.

**Table 3:** Results of hierarchical regression analyses.

	Self-objectification				Workplace anxiety			
	M1	M2	M3	M4	M5	M6	M7	M8
Gender	−0.12 (0.07)	−0.11 (0.07)	−0.10 (0.07)	−0.10 (0.07)	0.05 (0.10)	0.05 (0.10)	0.09 (0.07)	0.09 (0.07)
Age	−0.05 (0.04)	−0.072 (0.04)	−0.10^*^ (0.04)	−0.09 (0.04)	0.06 (0.06)	0.05 (0.06)	−0.02 (0.04)	−0.02 (0.04)
Education	0.09^*^ (0.04)	0.08 (0.04)	0.09 (0.04)	0.09 (0.04)	−0.05 (0.06)	−0.05 (0.06)	−0.02 (0.04)	−0.02 (0.04)
Organizational type	−0.01 (0.02)	−0.01 (0.02)	−0.01 (0.02)	−0.01 (0.02)	−0.020 (0.03)	−0.02 (0.03)	−0.04^*^ (0.02)	−0.04^*^ (0.02)
Organizational tenure	0.09^**^ (0.04)	0.10^**^ (0.04)	0.13^***^ (0.03)	0.12 (0.03)	−0.10^*^ (0.05)	−0.10^*^ (0.05)	−0.01 (0.04)	−0.02 (0.04)
Position	−0.08 (0.05)	−0.08 (0.05)	−0.09 (0.05)	−0.10 (0.05)	−0.10 (0.07)	−0.10 (0.07)	−0.12^*^ (0.06)	−0.12^*^ (0.06)
Work meaningfulness (WM)		0.13^**^ (0.04)	0.10^*^ (0.04)	0.10^**^ (0.04)		0.03 (0.06)	−0.08 (0.04)	−0.07 (0.04)
Performance pressure (PP)			0.24^***^ (0.04)	0.23^***^ (0.04)			0.76^***^ (0.05)	0.74^***^ (0.05)
PP^*^WM				0.08^*^ (0.04)				0.08^†^ (0.04)
R^2^	0.04	0.07	0.16	0.17	0.03	0.03	0.47	0.48
ΔR^2^		0.03	0.08	0.01		0.00	0.44	0.01
F	2.58^*^	3.83^**^	7.86^***^	7.54^***^	1.73	1.51	37.14^***^	33.72^***^
ΔF		10.88^**^	33.49^***^	4.34^**^		0.20	277.87^***^	3.86^***^

*** *p<* 0.001;

** *p<* 0.01;

* *p<* 0.05;

† *p=* 0.05.

#### Moderated Mediation

We tested the conditional indirect effect at two levels of work meaningfulness (i.e., +1 SD and−1 SD) by using MPLUS 7.4. The positive indirect effects of performance pressure on in-role behaviors through self-objectification were significant (*β*=0.04, *p*=0.02, 95% IC [0.01, 0.09]) when work meaningfulness was high but insignificant (*β*=0.02, *p*=0.18, 95% IC [0.001, 0.06]) when work meaningfulness was low. This difference was not significant (*β*=0.02, *p*=0.14, 95% IC [−0.001, 0.06]), thus the not supporting hypothesis 9. The negative indirect effect of performance pressure on in-role behaviors *via* workplace anxiety was significant (*β*=−0.12, *p*=0.01, 95% IC [−0.21, −0.04]) when work meaningfulness was high, and it was also significant (*β*=−0.11, *p*=0.01, 95% IC [−0.19, −0.04]) when work meaningfulness was low. The difference between these results was not significant (*β*=−0.02, *p*=0.30, 95% IC [−0.06, 0.002]), thus not supporting hypothesis 10.

## Discussion

### Theoretical Implications

Performance pressure is a double-edged sword, producing both helpful and harmful side effects for employees and organizations (Gardner, 2012; Gutnick et al., 2012; Mitchell et al., 2019). In this study, we use an approach/avoidance framework to gain a better understanding of the paradoxical effects of performance pressure on employees’ in-role behaviors. Our findings have several important implications for theory as following.

First, we found that performance pressure is indeed a double-edged sword toward employees’ in-role behaviors. On the one hand, it can activate employees’ sense of self-objectification, thereby enabling an approach motive that encourages the employees to become more engaged in their in-role behaviors. On the other hand, it can also engender workplace anxiety as an avoidance motive, which can distract employees from their tasks and hamper their capacity to perform in-role behaviors. Prior studies also confirmed that performance pressure is a double-edged sword, being appraised as a threat and challenge, and then eliciting functional and dysfunctional behaviors (Gardner, 2012; Gutnick et al., 2012; Mitchell et al., 2019). For instance, Gardner (2012) found that performance pressure produces both positive and negative outcomes within work teams. However, these literatures have not explored how employees react to this pressure based on an approach/avoidance motivation. Our study not only extend the understanding of the two-sided outcomes of performance pressure but also enrich the literature on performance pressure by indicating an application of the approach/avoidance model.

Second, we examined how self-objectification can serve to enable an approach motive that can mediate the positive indirect effects of performance pressure on employees’ in-role behaviors. Most previous studies have considered the role that objectification plays in the sexual realm (e.g., Tiggemann and Kuring, 2004; Szymanski and Henning, 2007; Andrighetto et al., 2017). And we extend objectification theory beyond the sexual realm to the work domain. Building on research on self-objectification in the work domain (e.g., Andrighetto et al., 2017; Caesens et al., 2017; Loughnan et al., 2017; Baldissarri et al., 2020), we show that self-objectification can have a positive effect on employees’ in-role behaviors, instead of only producing depression, aggression, or other negative effects (e.g., Jones and Griffiths, 2015; Poon et al., 2020). Therefore, we extend learning about objectification in the performance-based work setting and show the potentially positive side of self-objectification.

Third, we found that workplace anxiety can serve as an avoidance motive that mediates the negative indirect effect of performance pressure on an employee’s in-role behaviors. Anxiety is a prototypical avoidance motive, which commonly leads to withdrawal or submission in the face of stressors or negative stimuli (Lerner and Keltner, 2001; Ferris et al., 2016). Under performance pressure, employees often experience more anxiety and have greater difficulty paying attention to specific tasks, which results in diminished performance (McCarthy et al., 2016; Cheng and Mccarthy, 2018). We also answer the call issued by Cheng and Mccarthy (2018) for greater focus on understanding situational workplace anxiety. By drawing on attentional control theory (Eysenck et al., 2007), our findings extend learning about workplace anxiety as an avoidance motive.

Finally, we investigated the moderating role of work meaningfulness, and it is an influential factor that helps explain the approach and avoidance tendencies in response to performance pressure. Previous research has investigated many antecedents and outcomes of work meaningfulness (Rosso et al., 2010; Lysova et al., 2019), but few studies have identified the moderating role that work meaningfulness plays. The sense of work meaningfulness differs for each person, and analyzing this factor can help advance the understanding of the discrepancies between individual reactions to performance pressure. Therefore, we provide a broader understanding of work meaningfulness as a boundary condition.

### Managerial Implications

Performance pressure is designed to improve an organization’s capacity to achieve its goals, and such pressure is important because an organization’s success depends on its employees’ productivity (Mitchell et al., 2019). Our results show that performance pressure is a double-edged sword, motivating an approach (i.e., self-objectification) and avoidance (i.e., workplace anxiety) toward employees’ in-role behaviors. Therefore, we suggest that managers should be aware of its two-sided nature. From the employee’s perspective, managers should convey performance expectations that are both challenging and reasonable. They should clearly communicate the benefits and opportunities involved in attaining better performance, as a mean to accentuate the positive and attractive effects of pressure while avoiding the negative effects. From the manager’s perspective, performance pressure may also generate temptations for the managers to commit unethical acts like earning management or sabotage (Zhou et al., 2020), either because positive incentives (motivation to receive a performance bonus for example) or because negative incentives (fear to be fired). Thus, organizations should value and appropriately apply performance pressure.

Another implication for managers is that it is important to emphasize the value of work meaningfulness. We show that high levels of work meaningfulness can inspire employees to pay more attention in their work and to approach rather than avoid the challenge of performance pressure. Work meaningfulness is critical to determining how employees approach, formulate, and experience their work and their workplace (Brief and Nord, 1990; Rosso et al., 2010). Lysova et al. (2019) also emphasized that fostering meaningful work is a significant means of attracting employees. Therefore, leaders should shape meaning and belief regarding work by framing the mission, goals, purposes, and importance of the tasks that employees do (Podolny et al., 2005; Rosso et al., 2010). This approach can activate employees’ tendencies to voluntarily objectify themselves and to work hard for goals they support. In addition, it should be noted that employees with high levels of work meaningfulness can experience more anxiety in response to performance pressure. As an old saying goes, caring too much can lead to worry and anxiety. Thus, to enable the constructiveness of their employees’ interpretations, communications, and responses, managers need to weaken their employees’ tendencies to avoid challenges while seeking to reduce their concerns.

### Limitations and Future Research Directions

We acknowledge the limitations of our study. First, our research conducted formal test without pre-testing the questionnaire. The scales are recognized mature scales and widely used home and abroad with higher internal consistency and retest reliability, but the validity of the instrument remains a concern. In the future, such kind of research should pre-test the questionnaire before data collection. Second, we collected data from a single source self-reported due to resource constraints plus during the COVID-19 pandemic may promote CMV bias (Podsakoff et al., 2003, 2012). Further, we conducted a statistical procedural remedy as proposed by Podsakoff et al. (2003), and we were able to demonstrate that common method bias was not a serious issue in this study. And we also conducted multilevel confirmatory CFA and showed that our measurement model has a better fit to the data (*χ*^2^[df]=536.07 [199], CFI=0.92, RMSEA=0.07, SRMR=0.07) than any other alternative models. Even so, future studies should make efforts to reduce the likelihood of common method bias by conducting cross-lagged designs, collecting multisource data, and conducting longitudinal studies. Last, our study failed to determine causality. The cross-sectional nature of our empirical data signified reverse causality, which can be determined through experimental manipulation (Ferris et al., 2016). We hope that our theoretical construction can support the inference of causality. An approach/avoidance framework presumes that the presence of stimuli produces movement either toward or away from stimuli (Elliot, 2006; Ferris et al., 2016). Accordingly, we considered performance pressure as one such stimulus for employees (Gutnick et al., 2012; Mitchell et al., 2019), which subsequently arouses distinctive reactions, namely of either approach toward or avoidance away from the desired in-role behaviors. Our results were consistent with this causal logic, but they did not definitively demonstrate it. Thus, we call for future studies to combine experimental and empirical studies to explain the causation involved more rigorously.

## Conclusion

We demonstrate that performance pressure is a double-edged sword for employees. The findings suggest that performance pressure can activate employees’ self-objectification, thereby encouraging them to become more engaged in their in-role behaviors. However, such pressure can also produce workplace anxiety, thereby hampering employees’ in-role behaviors. Work meaningfulness influences how employees experience performance pressure. Employees with a strong sense of work meaningfulness are more capable of self-objectification and of coping with performance pressure and employees also feel more anxious under performance pressure due to too much concern. We hope that our study helps theorists and business leaders better understand performance pressure and its relevant consequences.

## Data Availability Statement

The raw data supporting the conclusions of this article will be made available by the authors, without undue reservation.

## Author Contributions

XX, YW, and ML conducted data collection. XX wrote the first draft, HKK gave direction and granted financial support. All authors (including XX, YW, ML, and HKK) commented on previous versions of the manuscript and approved the final manuscript.

## Conflict of Interest

The authors declare that the research was conducted in the absence of any commercial or financial relationships that could be construed as a potential conflict of interest.

## Publisher’s Note

All claims expressed in this article are solely those of the authors and do not necessarily represent those of their affiliated organizations, or those of the publisher, the editors and the reviewers. Any product that may be evaluated in this article, or claim that may be made by its manufacturer, is not guaranteed or endorsed by the publisher.
